# Identification of a novel *DMD* duplication identified by a combination of MLPA and targeted exome sequencing

**DOI:** 10.1186/s13039-017-0301-0

**Published:** 2017-03-23

**Authors:** Beibei Wu, Liying Wang, Ting Dong, Jiahui Jin, Yili Lu, Huiping Wu, Yue Luo, Xiaoou Shan

**Affiliations:** 1 Department of Pediatrics, The Second Affiliated Hospital and Yuying Children’s Hospital of Wenzhou Medical University, 109 West Xueyuan Road, Wenzhou, Zhejiang 325027 People’s Republic of China; 2 Capital Medical University, Beijing, 100069 China

**Keywords:** Duchenne muscular dystrophy, Dystrophin, Targeted exome sequencing, MLPA, Duplication

## Abstract

**Background:**

Duchenne muscular dystrophy (DMD) is an X-linked recessive muscle-wasting disease caused by a mutation in the *DMD* gene. The aim of this study was to identify a de novo mutation of the *DMD* gene in the family of a 9-month-old Chinese male patient, as well as to describe the phenotypic characteristics of this patient.

**Results:**

The patient was suspected to suffer from DMD according to physical examination, biochemical analyses, and electromyogram. We identified a duplication of exons 4–42 in *DMD* gene with targeted exome sequencing and multiplex ligation-dependent probe amplification (MLPA). In addition, the patient’s mother was a carrier of the same mutation.

**Conclusions:**

We identified a de novo duplication of exons 4–42 in a patient with early stage DMD. The discovery of this mutation may provide insights into future investigations.

## Background

Duchenne muscular dystrophy (DMD), an X-linked recessive muscle-wasting disease, is caused by a mutation in the *DMD* gene that encodes the dystrophin protein [1, 2]. As the most common muscle disease in children, the incidence of DMD is 1 in 3500 live male births [3]. DMD is a progressive neuromuscular disease characterized by pseudohypertrophy in the calf muscle and the Gowers’ sign [4]. Patients with DMD are usually first diagnosed before 5 years of age and many pass away due to respiratory or cardiac failure at around 20 years of age [5, 6].

DMD is the largest known human gene encompassing 2.2 Mb of nucleotides that contain 79 exons [6]. The large size of the gene increases its susceptibility to mutations as evidenced by the one-third of de novo mutations identified in DMD [4]. Furthermore, intragenic deletions represent 65–70% of all mutations, while duplications are found in 7% of patients and the remaining mutations are either point mutations or small deletions/insertions [4].

Diagnosis of the DMD mainly depends on the genetics of the patient [4]. Recent studies showed that targeted exome sequencing is a powerful tool for diagnosis of Mendelian diseases [7], This is especially true for patients who are at the early stage of a disease and show no obvious clinical manifestation. Targeted exome sequencing can screen for mutations in a panel of many genes that are implicated in various inherited diseases, making it a tremendously useful technique for identification of disease associated mutations.

However, targeted exome sequencing is relatively less powerful in testing large intragenic rearrangements. In the case of DMD, deletions found in most patients are followed by duplications, therefore, multiplex PCR, which can detect up to 98% of deletion, was considered as the standard diagnostic method [8]. Nevertheless, multiplex PCR is not the ideal technique for identifying duplications and female carriers due to the presence of a normal copy of the *DMD* gene [3]. Fortunately, the introduction of a dosimetry method based on multiplex ligation-dependent probe amplification (MLPA) has improved the discovery of large intragenic rearrangements [8].

In this study, we implemented a combination of MLPA with targeted exome sequencing to comprehensively screen for large fragment duplications in the *DMD* gene in a 9-month-old Chinese male patient. Using this method, we were able to identify a novel duplication of exons 4–42 in the *DMD* gene.

## Methods

### Patient characteristics

This study was in compliance with the Declaration of Helsinki and was approved by the Ethics Committee of The Second Affiliated Hospital and Yuying Children’s Hospital of Wenzhou Medical University. A 9-month-old male patient was referred to our hospital in 2015. Written informed consent was obtained from his statutory guardian. Patients’ initial symptoms and complaints was diarrhea. The patient received a comprehensive examination including physical examination, biochemical analyses and electromyogram. Detailed family history was obtained from the patient, and peripheral blood samples were collected.

### MLPA

MLPA analysis was performed using SALSA MLPA kit P034/P035 (MRC-Holland, Amsterdam, the Netherlands) kit according to manufacturer’s instructions. The MLPA samples consisted of approximately 100 ng of genomic DNA. Denaturation, hybridization, ligation and amplification were performed with ABI 2720 PCR thermal cycler. The PCR amplification was performed under the following conditions: 33 cycles of 95 °C for 30 s, 60 °C for 30 s and 72 °C for 60 and a final extension step at 72 °C for 20 min to allow for adequate probe hybridization with the two sets of probes. Results were analyzed using Coffalyser 9.4 (MRC Holland).

### Targeted exome sequencing

Genomic DNA was extracted from blood samples using a DNA Extraction kit (TIANGEN, Beijing) according to the manufacturer’s instructions. The coding exons of 131 genes related to inherited diseases were selected and captured with a GenCap custom enrichment kit (MyGenostics, Beijing) as previously described [9]. Genomic DNA was fragmented and mixed with GenCap probe (MyGenostics, Beijing) for PCR amplification and hybridization. After washing with MyOne beads (Life Technology), the sample was resuspended in binding buffer, transferred along with MyOne beads, and rotated for 1 h. Then, DNA was eluted with Buffer Elute, followed by amplification in post-capture. The enriched libraries were sequenced on a IlluminaSolexaHiSeq 2000 sequencer. After removing PCR duplicates with Picard program, the clean reads were aligned with SOAP aligner program [10] according to human genome parameters (hg19). Then, we identified SNPs using the SOAPsnp program, realigned the reads by BWA, and determined the deletions or insertions (InDels) with the GATK software [11]. After annotation of the identified InDels and SNPs using the Exome-assistant program (http://122.228.158106/exomeassistant), candidate SNPs, InDels and the short read alignments were viewed by Magic Viewer [12] To determine pathogenicity, four algorithms, PolyPhen, SIFT, Mutationtaster and PMut were used to evaluated nonsynonymous variant. Sequencing data were deposited in to NIH Short Read Archive (SRP033329).

## Results

**Fig. 1:**
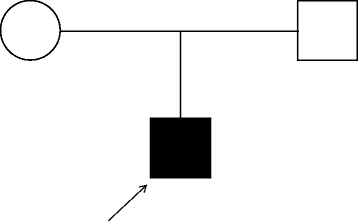
Pedigree analysis. The patient is demarcated in black. The patient was homozygous for the mutation and his mother who had the same genotype was a carrier of DMD

The 9-month-old male patient was diagnosed with DMD. The patient was an only child of his healthy parents. There was no medical history of note (Fig. 1). The main complaint of the patient was diarrhea. Physical examination revealed no obvious signs or symptoms. Biochemical analyses showed an increased level of creatine phosphokinase (CPK), which indicated that the patient suffered from muscle damage. Increased alanine aminotransferase (ALT) and aspartate aminotransferase (AST) suggested liver dysfunction. In addition, elevated LDH revealed tissue damage (Table 1). We also performed electromyogram on the patient, which revealed that rectus femoris and bicep muscles were showed mix type when at maximum average unit width (Table 2). Result of electromyogram suggested myogenic damage.

**Table 1:** Results of patient biochemical test

Parameter	Test value	Change	Reference range
CPK, U/L	12517	↑	38–174
ALT, U/L	107	↑	9–50
AST, U/L	193	↑	15–40
LDH, U/L	773	↑	109–245

CPK, creatine phosphokinase

**Table 2:** Results of patient electromyogram

Muscle	maxium average unite width
Left rectus femoris	mix type
Right rectus femoris	mix type
Left biceps	mix type
Right biceps	mix type

**Fig. 2:**
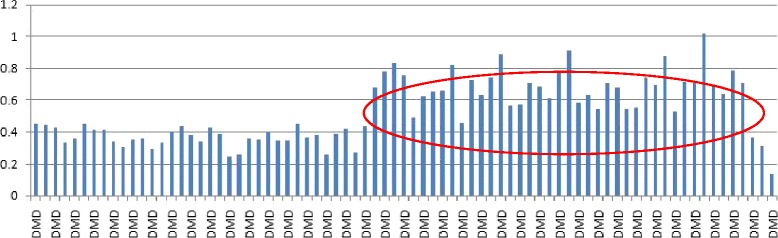
Target exome sequencing of the proband. The results indicated there was a potential large duplication in the *DMD* gene

We used a panel of 131 inherited disease-causing genes to screen for mutations. Our targeted exome sequencing reached an average mean depth of 168X with greater than 97.8% coverage of the targeted regions. After alignment and bioinformatics analyses, single nucleotide variants (SNVs) and insertions/deletions (Indels) were annotated to the exome database, among which those with MAF >0.005 or homozygosity of >1 were filtered out. The missense variants were discarded based on tolerant prediction using in silico tools. Although we failed to detect any point mutation, our results indicated a potential large duplication in the *DMD* gene (Fig. 2).

**Fig. 3:**
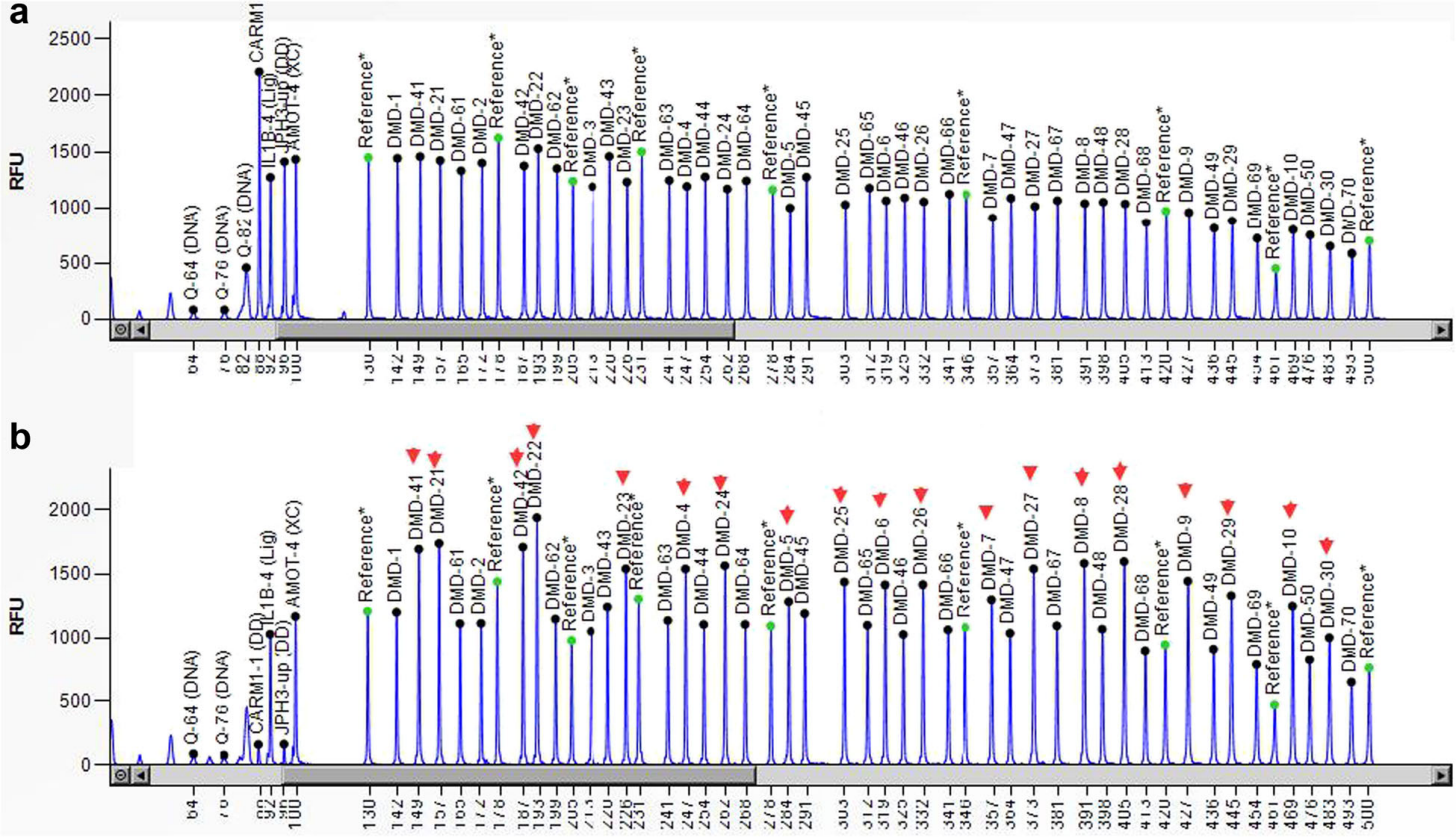
MPLA results of the proband. **a** Normal control. **b** MPLA identified the patient duplication of exons 4–42, which marked as arrows is a a de novo mutation

**Fig. 4:**
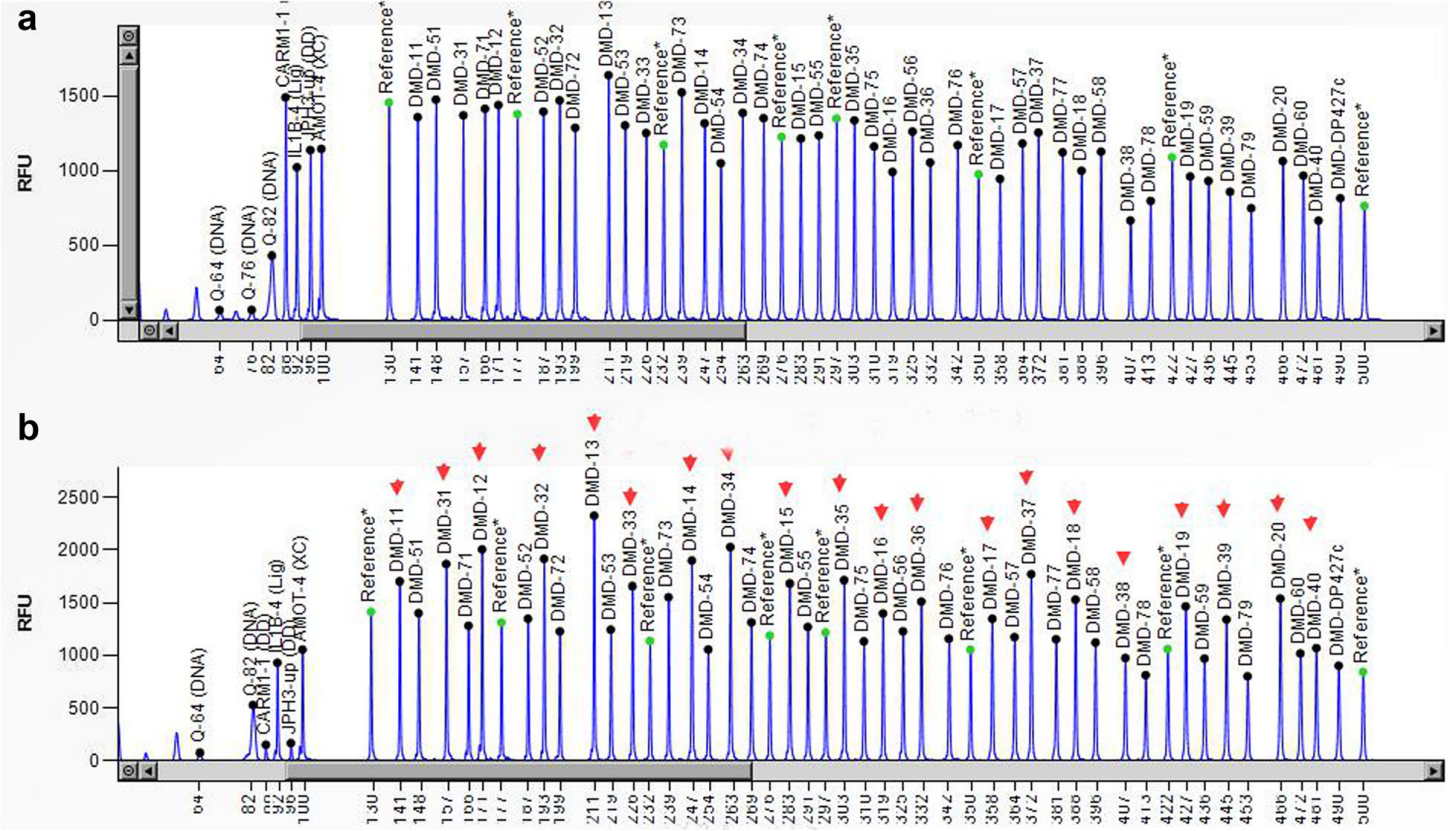
MPLA results of the patient’s mother. **a** Normal control. **b** The identified mutations in the mother of patient was duplication of exons 4–42, which were marked as arrows. The patient’s mother was a carrier who had the same mutation

In order to identify the precise mutation site, we performed MLPA to detect complex rearrangements in the patient. Results from MLPA analysis showed a novel duplication involving exons 4–42 in the *DMD* gene (Fig. 3). Since DMD is an X-linked recessive muscle-wasting disease, pedigree analysis further indicated that the mother is a carrier of this mutation (Fig. 1). We, then, performed MLPA analysis on the mother of the patient and identified that the she does, in fact, have duplication of exons 4–42, the same genotype as the patient (Fig. 4). This mutated rearrangement has not been previously reported in the Leiden database (www.dmd.nl), and therefore, the genotype-phenotype correlation remained unknown.

## Discussion

In this study, we described a DMD family with a de novo duplication of exons 4–42, first suggested by targeted exome sequencing and subsequently detected by MLPA analysis. The patient was a 9-month-old male that did not show any typical characteristics of DMD, such as calf muscle pseudohypertrophy, Gowers’ sign. Biochemistry tests showed sharply increased CPK and electromyogram suggested myogenic damage, which led us to speculate that the patient suffers from DMD. However, clinical evidence was not sufficient for a formal diagnosis of DMD. In addition, increased level of ALT, AST and LDH suggested liver damage. Therefore, differential diagnosis of diseases that could lead to liver damage, such as viral hepatitis and Wilson’s disease, should be investigated. We, therefore, performed a viral hepatitis serology screen and tested for ceruloplasmin in serum of the patient. The results of viral hepatitis serology screen were negative, which ruled out viral hepatitis. In addition, the level of ceruloplasmin was normal, which did not support a diagnosis of Wilson’s disease.

Genetic testing is the mainstay of diagnosis for DMD [4]. Despite that MLPA is now the most widely used technique for identifying duplication in DMD [8], targeted exome sequencing is prefered when evidence for a diagnosis of DMD is insufficient and when other metabolic diseases have not been ruled out. Furthermore, targeted exome sequencing is capable of detecting subtle mutations in the 79 exons that cannot be detected by MPLA [3]. In our patient, results from targeted exome sequencing were suggestive of a potential duplication in the *DMD* gene. We then confirmed this finding using MLPA. A combination of MLPA with targeted exome sequencing can therefore, be a valuable method for identifying *DMD* gene defects.

Dystrophin, as encoded by the *DMD* gene, is predominantly expressed in skeletal and cardiac muscles [6]. As a significant component of the dystrophin-glycoprotein complex (DGC), dystrophin, plays a key role in the contraction of sarcomeres [13]. Dystrophin is a structural protein of 427-kD, consisting of a N-terminal actin binding domain (ABD1), a central rod region, a cysteine-rich (CR) globular domain, and a C-terminal tail (CT) [13]. The C-terminus is associated with glycoproteins and the N-terminus is associated with actin or actin-like proteins [1]. Previous study suggested that dystrophinis is a cytoskeletal protein that is potentially involved in linking the extracellular matrix to the interior of muscle fibers. Mutations in the *DMD* gene, therefore, may disrupt such linkage and ultimately lead to DMD as a result of sarcolemma instability [1]. As the disease progresses, DMD patient may lose the ability to walk without assistance and may eventually die from respiratory or cardiac failure as a result of relative muscle damage [14].

The only pharmacologic agent proven to be effective for treating DMD is corticosteroids. Long-term studies indicated that long-term daily administration of corticosteroids reduces the decline of cardiorespiratory function, lowers the risk of progressive scoliosis, prolongs ambulation by up to 3 years, and improves life expectancy of DMD patients [15–18]. In addition, novel therapies for DMD, such as stem cell therapy, gene therapy (e.g. viral vectors), and dystrophin restoration approach, have recently been developed [4]. In this study, we demonstrated that a combination of targeted exome sequencing and MLPA is a valuable diagnostic method for detecting DMD at the early stage of the disease, which is critical for early intervention and can ultimately lead to better prognosis of DMD patients. If a patient with rapid increase of CPK, even though does not show any typical characteristics of DMD, such as calf muscle pseudohypertrophy, Gowers’ sign, we should suspect DMD and consider to perform Targeted exome sequencing and MLPA.

## Conclusions

In summary, our study identified a de novo duplication of exons 4–42 in the DMD gene in a 9-month-old male Chinese patient using a combination of targeted exome sequencing and MLPA. The discovery of this novel mutation may provide important insights for future investigations.
